# Effective treatment for suppression of acrylamide formation in fried potato chips using L-asparaginase from *Bacillus subtilis*

**DOI:** 10.1007/s13205-015-0278-5

**Published:** 2015-02-04

**Authors:** Yohei Onishi, Asep A. Prihanto, Shigekazu Yano, Kazuyoshi Takagi, Midori Umekawa, Mamoru Wakayama

**Affiliations:** 1 Department of Biotechnology, Faculty of Life Sciences, Ritsumeikan University, 1-1-1 Nojihigashi, Kusatsu, Shiga 525-8577 Japan; 2 Department of Biochemical Engineering, Graduate School of Sciences and Engineering, Yamagata University, Jonan, Yonezawa, Yamagata 992-8510 Japan; 3 Department of Applied Chemistry, Faculty of Life Sciences, Ritsumeikan University, 1-1-1 Nojihigashi, Kusatsu, Shiga 525-8577 Japan

**Keywords:** Asparaginase, *Bacillus subtilis*, Acrylamide, Mitigation, Potato chips

## Abstract

It has been reported that acrylamide, a potential carcinogen, is formed from the reaction of L-asparagine (L-Asn) and reducing sugars contained in foods during heating processes and free asparagine is a limiting factor for acrylamide formation. It has been reported that potato products such as potato chips, which are made through heating processes, contain high levels of acrylamide. To decrease the amount of L-Asn in potatoes using L-asparaginase, effective treatment conditions of sliced potatoes with the enzyme have been investigated. By treating sliced potatoes with *Bacillus subtilis* L-asparaginase II (BAsnase; 4 U/g potato), appriximately 40 % of L-Asn in the sliced potatoes was converted into L-aspartic acid (L-Asp). To make this enzyme more effective, prior to enzymatic treatment, sliced potatoes were freeze-thawed, dried at 90 °C for 20 min, and vacuum treated for 10 min under decompressed condition, resulting in the hydrolysis of approximately 90 % of L-Asn to L-Asp. The acrylamide content of BAsnase-treated fried potato chips decreased to below 20 % of that of BAsnase-untreated fried potato chips. Treatment conditions examined in this study were found to be effective to suppress the formation of acrylamide in fried potato chips.

## Introduction

Several agricultural products are treated by heating such as frying, baking, roasting, steaming, and boiling before being sold in the markets. French fries, potato chips, bread, and coffee beans are typical commercial agricultural products sold in the markets after heat treatment. On the other hand, it has been confirmed that a wide range of foods prepared by heating treatments like fried potatoes, coffee, bread, and biscuit contain high levels of acrylamide, a potential human carcinogen (Rosen and Hellenas 2002). Therefore, it is important to reduce the acrylamide content to moderate levels, which could correspond to a negligible cancer risk in these products (Tareke et al. 2002). The possible mechanisms of acrylamide formation in processed foods have recently been the focus of intense research. The formation of acrylamide via a Maillard reaction has been reported (Mottram et al. 2002; Stadler et al. 2002). A mechanism of acrylamide formation by the reaction of asparagine and a carbonyl-containing compound, such as reducing sugars, at typical cooking temperatures has been proposed (Zyzak et al. 2003; Yaylayan et al. 2003; Becalski et al. 2003).

For example, both cereal- and potato-based food products that have been prepared via heating processes contain high amounts of acrylamide (30–5,600 ng/g) (Tareke et al. 2002; Becalski et al. 2003; Tsutsumiuchi et al. 2004). Pretreatment recipes and strategies to reduce acrylamide formation have been proposed, i.e., changing the time and temperature for heat, using potato with low asparagine content, controlling the level of reducing sugars and amino acid content by blanching and immersing into water, and adjusting the pH (Becalski et al. 2003; Amrein et al. 2003; Rydberg et al. 2003; Rommens et al. 2008). Prolonged blanching and immersing have been proved to be effective for the reduction of acrylamide formation (Pedreschi et al. 2006). However, these treatments could suppress not only the acrylamide formation but also the formation of desired Maillard products, thus having a negative impact on both taste and appearance of the product.

L-Asparaginase (L-asparagine amidohydrolase: EC 3.5.1.1) catalyzes the hydrolysis of L-Asn to L-Asp and ammonia and is present in various organisms from microorganisms to mammals. Two types of L-asparaginases have been reported, namely L-asparaginase I with a low affinity for L-asparagine and L-asparaginase II with a high affinity for L-asparagine (Cedar and Schwarts 1967). In particular, the type II enzymes of *Escherichia coli* and *Erwinia chrysanthemi* have been investigated with respect to their antitumor activity and effectively employed as therapeutic drugs for acute lymphoblastic leukemia (Clavell et al. 1986). Recently, fungal L-asparaginases have been also investigated as anticancer drugs (Theantana et al. 2009). On the other hand, few studies have reported the use of microbial asparaginases in the food industry.

L-Asparaginase is considered to be useful for acrylamide mitigation and to have negligible effects on the general formation of Maillard products. L-Asparaginase can selectively reduce the level of free L-Asn by hydrolyzing it to L-Asp and ammonia, thereby specifically removing one of the essential acrylamide precursors. The effective use of L-asparaginase for acrylamide mitigation has been demonstrated using asparaginase II from *E. coli*, which was originally developed as a pharmaceutical agent (Zyzak et al. 2003; Amrein et al. 2003). To the best of our knowledge, for the first time asparaginase from *Aspergillus oryzae* has been recently reported as the enzyme from food-grade microorganism (Pedreschi et al. 2008; Hendriksen et al. 2009). Those studies demonstrated that the application of L-asparaginase in food processing resulted in a reduction in their asparagine levels and subsequently the lower acrylamide levels in the final products when compared with untreated products.

*Bacillus subtilis* has been widely used in the fermented food industry in Asian countries and its safety in food production has also been verified. *B. subtilis* has two L-asparaginase genes, *ans*A and *ans*Z. We reported the cloning of *ans*A and *ans*Z and the characterization of their products corresponding to L-asparaginase I and L-asparaginase II, respectively (Yano et al. 2008; Onishi et al. 2011; Jia et al. 2013), indicating that L-asparaginases from *B. subtilis* can be useful for acrylamide mitigation by reducing L-Asn. This paper describes and discusses the application of *B. subtilis* L-asparaginase II (BAsnase) to fried potato chips production.

## Materials and methods

### Materials

BAsnase was prepared according to the procedure described previously (Onishi et al. 2011). The expression plasmid was constructed by inserting *ans*Z gene for *N*-terminal signal sequence truncated enzyme into pET-21a at the restriction sites of *Nde*I and *Bam*HI. *Escherichia coli* Rosetta-gami B transformed with the plasmid was cultured in LB medium in the presence of isopropyl β-d-thiogalactoside and antibiotics such as ampicillin, tetracycline, and chloramphenicol at 30 °C for 20 h with continuous shaking. Recombinant *E. coli* cells were suspended in 10 mM potassium phosphate buffer (pH 8.0), and disrupted by sonication. The supernatant dialyzed against the same buffer was used as a crude BAsnase for further purification. BAsnase was purified by two-step column chromatography, DEAE-Toyopearl and Butyl-FF to electrophoretic homogeneity. The purified enzyme with a specific activity of 45.4 U/mg was used in this study. L-Asparagine (L-Asn) and L-aspartic acid (L-Asp) were purchased from Wako Pure Chemicals (Tokyo, Japan). All other chemicals were of analytical grade. Potatoes grown in Hokkaido, Japan, which were used for potato chips, were purchased in a grocery store.

### Enzyme and protein assay

The enzyme activity was measured by determining the amount of ammonia formed with glutamate dehydrogenase (GLDH) (Yano et al. 2008). The reaction mixture contained 100 mmol/l potassium phosphate buffer (pH 8.0), 30 mmol/l, l-Asn, and the enzyme in a final volume of 1 ml. After being allowed to proceed for 10–20 min for 30 °C, the reaction was terminated by boiling for 3 min. The reaction mixture (50–100 μl) was transferred to a GLDH reaction mixture containing 100 mmol/l Tris–HCl buffer (pH 8.0), 2.4 mmol/l NADH, 100 mmol/l 2-oxoglutarate, and 5 units of GLDH in a total volume of 1 ml. The absorbance change at 340 nm was measured after incubating the mixture at 30 °C for 60 min. One unit of the enzyme was defined as the amount of enzyme that catalyzed the formation of 1 μmol ammonia per min. Protein concentration was determined using the Lowry method with crystalline bovine serum albumin as the standard (Lowry et al. 1951).

### Preparation of sliced potato and fried potato chips

After washing the surface of the potatoes and measuring their weights, they were peeled and cut into four pieces in approximately equal size. Each piece of potato was further cut into small pieces with the thickness of approximately 1.5 mm and the moisture on the surface was dried off using filter papers. Small pieces of sliced potato weighing in the range of 10–15 g were transferred in petri dishes without any bias. Sliced potatoes with or without treatments described below were fried at 170 °C for 90 s in corn oil (Nisshin Co.). After frying, the excess amount of oil was removed by placing them on filter papers for 5 min.

### Treatment of sliced potato with BAsnase

The effect of BAsnase treatment of sliced potatoes on the reduction of L-Asn was examined. BAsnase (0–40 U) was spread evenly by applying it on one surface of the sliced potato. Then, sliced potatoes were kept in the incubator at 60 °C for 10 min. The extract of sliced potatoes was prepared according to the procedure described in the section, “Preparation of sample extract”.

### Pretreatment of sliced potato prior to BAsnase treatment

Three different pretreatments were performed.

1. Drying treatment of sliced potatoes: sliced potatoes were treated at 90 °C for 0, 10, and 20 min in the dryer.
2. Vacuum treatment of sliced potatoes: sliced potatoes were treated at room temperature for 10 min under decompressed condition produced by an aspirator (3.2 × 10^−3^ – 1.6 × 10^−3^ MPa, EYELA A1000S).
3. Freezing treatment of sliced potatoes: sliced potatoes were treated at −20 °C for 20 min in the freezer and then thawed at room temperature. After each treatment described above or combination of treatments (Fig. 3a–e), 40 U of BAsnase was spread evenly by applying it on one surface of the sliced potato and incubated at 60 °C for 10 min. The sliced potato extract was also prepared using the same procedure described above.

### Preparation of sample extract

The sliced potato samples (10–15 g) were homogenized with a blender. The homogenate was added with 70 ml of 75 % ethanol (Amrein et al. 2003). The homogenate was mixed for 7 min with a 30-s interval. A soluble fraction was recovered by filtration. To an insoluble fraction, 20 ml of 75 % ethanol was added and mixed to create a homogeneous mixture using the same procedure. After recovering a soluble fraction by filtration, 10 ml of 75 % of ethanol was added to the unrecovered homogenate, and a soluble fraction was recovered. All the recovered soluble fractions were collected and filtrated using a 0.45-μm pore-size membrane (ADVANTEC, Tokyo, Japan) and then used for the analysis of acrylamide and amino acids.

### Analysis of acrylamide

Sample extract was sent to Shimadzu Analytical Center Co. for acrylamide analysis. Analysis of acrylamide in the sample extract was performed using GCMS-QP2010 (Shimadzu Co., Kyoto, Japan) with acrylamide (^13^C_3_) as an internal standard.

### HPLC analysis

The extract of sliced potato was filtrated using 0.45 µm pore-size membrane before derivatization. Amino acids in the sample were derivatized to a fluorescent compound using *o*-phthaldialdehyde (OPA) and *N*-*tert*-butyloxycarbonyl-L-cysteine (BOC) and analyzed by reversed–phase HPLC as follows: 5 µL of the filtered sample was mixed with 100 µL of OPA-Boc solution prepared by dissolving 10 mg OPA and 10 mg Boc in 1 mL methanol and 395 µL of 0.4 M borate buffer (pH 9.0). The mixture was incubated at room temperature for 10 min and 5 µL of the mixture was applied to HPLC. HPLC analysis was performed using a Cosmosil 5C18-MS-II column (4.6 × 250 mm; Nacalai Tesque, Kyoto, Japan) and equilibrated with 0.1 M acetate buffer (pH 6.0) containing 7 % acetonitrile and 3 % tetrahydrofuran. The derivatized amino acids were eluted using a linear gradient of acetonitrile (7–47 %). The elute was monitored by measuring its fluorescence at excitation and emission wavelengths of 344 and 433 nm, respectively, and identified by their respective retention time using L2480 Fluorescence Spectrophotometer (Hitachi, Tokyo, Japan).

## Results

### Effect of BAsnase treatment on L-Asn reduction

**Fig. 1:**
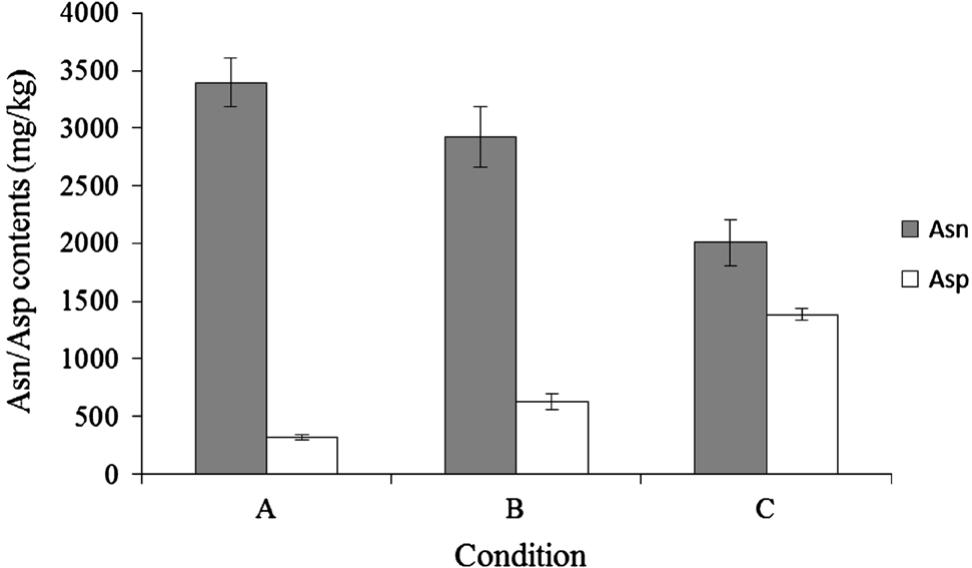
Effect of BAsnase treatment on L-Asn and L-Asp contents of sliced potatoes. Sliced potatoes were incubated at 60 °C for 10 min with or without BAsnase. **a** Without enzyme, **b** with 10 U; **c** with 40 U. The values represent the mean ± SD (*n* = 5)

To examine the effect of BAsnase on the reduction of L-Asn in sliced potatoes, the enzyme (10–40 U) was evenly applied on the surface of the sliced potatoes with a thickness of 1.5 mm. After incubation at 60 °C for 10 min, the sliced potato extract was prepared as described in the “Materials and methods”. Concentrations of L-Asn and L-Asp were determined by HPLC. As shown in Fig. 1, the L-Asn content in sliced potatoes decreased after the treatment with BAsnase proportionally to the enzyme concentration, thus suggesting that BAsnase is effective for the reduction of l-Asn content in sliced potatoes.

### Effect of drying, vacuum and freezing treatments prior to BAsnase treatment on L-Asn reduction

The effects of the pretreatment on the sliced potatoes before BAsnase treatment on the reduction of L-Asn were examined. First, the sliced potatoes were treated in a dryer box at 90 °C for 10–20 min, and then 40 U of BAsnase was evenly applied onto the surface of the sliced potatoes and they were incubated at 60 °C for 10 min. After enzyme treatment, concentrations of L-Asn and L-Asp of the sliced potatoes were determined. Consequently, the drying treatment of the sliced potatoes was not effective independently as a pretreatment (data not shown).

**Fig. 2:**
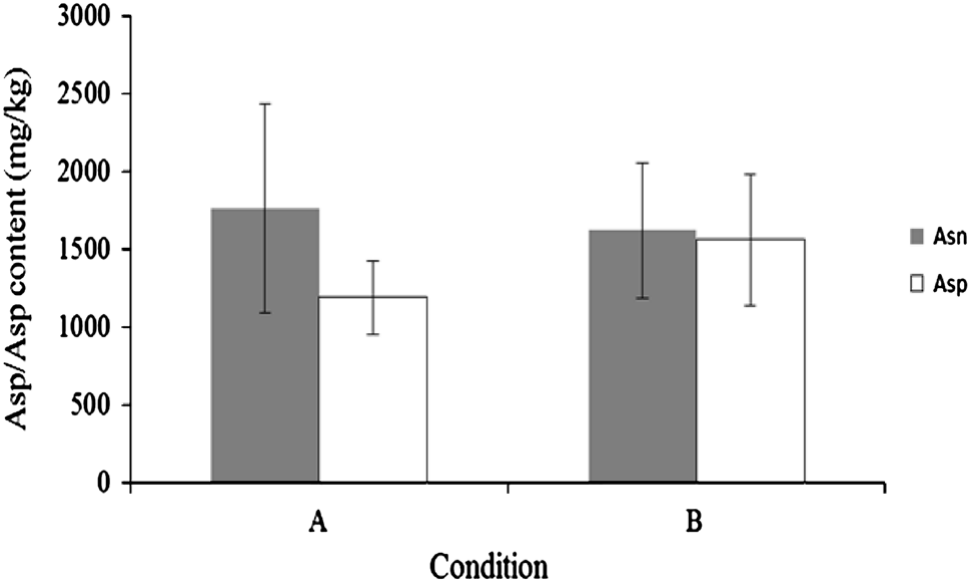
Effect of reduced-pressure pretreatment on conversion of L-Asn to l-Asp by BAsnase. Sliced potatoes were incubated at 60 °C for 10 min with 40 U of BAsanse after treatment of **a** or **b**. **a** No reduced-pressure pretreatment, **b** reduced-pressure pretreatment. The values represent the mean ± SD (*n* = 5)

**Fig. 3:**
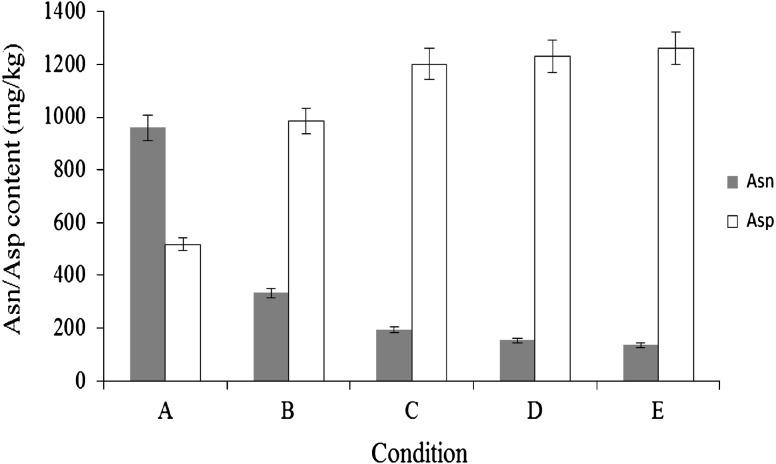
Effect of combination pretreatments on conversion of L-Asn to L-Asp by BAsnase. Sliced potatoes were incubated at 60 °C for 10 min with 40 U of BAsanse after treatment of **a**–**e**. **a** No pretreatment, **b** freezing and thawing, **c** drying after freezing and thawing, **d** reduced-pressure pretreatment after freezing and thawing, **e** reduced-pressure pretreatment following drying after freezing and thawing. The values represent the mean ± SD (*n* = 5)

After keeping the sliced potatoes under vacuum condition for 10 min at 30 °C, 40 U of BAsnase was evenly applied onto the surface of the sliced potatoes and they were incubated at 60 °C for 10 min. After the enzyme treatment, concentrations of L-Asn and L-Asp were determined. As shown in Fig. 2, treating sliced potatoes under vacuum conditions resulted in a slightly positive effect independent of the reduction of L-Asn. After keeping the sliced potatoes at −20 °C for 20 min, and thawing them at room temperature, 40 U of BAsnase was evenly applied onto the surface of the sliced potatoes and they were incubated at 60 °C for 10 min. As shown in Fig. 3, the freezing before the enzyme treatment was much more effective than the enzyme treatment alone. The effect of the combination of freezing with the other treatments was examined on the reduction of L-Asn. The optimal condition for the reduction of L-Asn in potato chips production was determined as follows: freezing sliced potatoes at −20 °C for 20 min, thawing them at room temperature for 20 min, drying them at 90 °C for 10 min, treating them under vacuum condition for 10 min, and treating them with 40 U of BAsnase for 10 min at 60 °C. Drying and vacuum treatment in addition to freezing as pretreatments resulted in a considerable positive effect on the reduction of L-Asn. The series of treatments were considered to allow the enzyme to permeate into the sliced potatoes. The L-Asn content of sliced potatoes treated under optimal conditions decreased to approximately one-tenth of that of the untreated sliced potatoes (Fig. 3).

### Effect of pretreatments on acrylamide reduction in fried potato chips

**Fig. 4:**
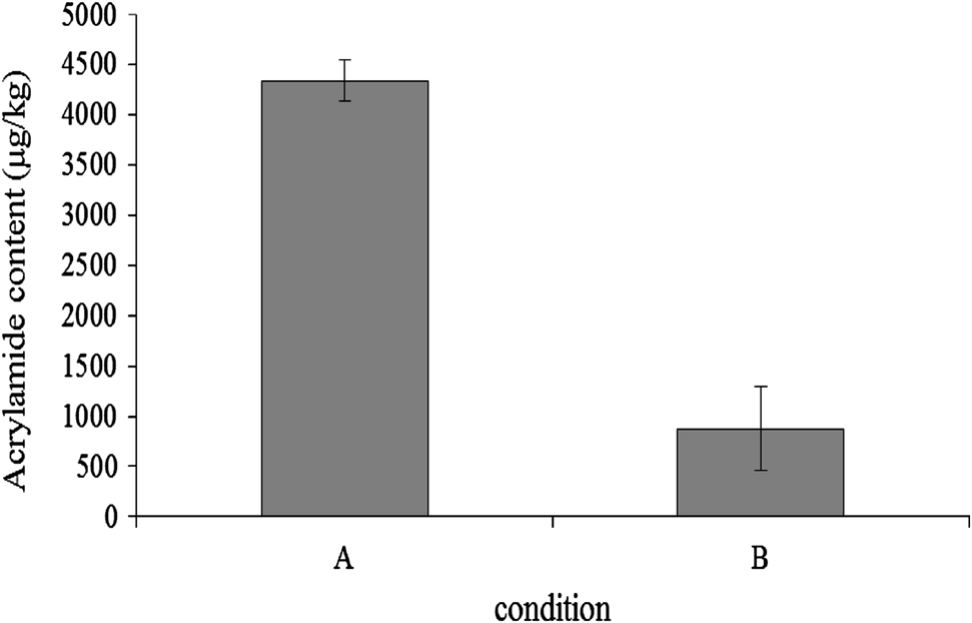
Effect of BAsnase treatment on acrylamide content of fried potato chips. **a** Without any treatments, **b** with enzyme treatment following pretreatments (condition **e** described in Fig. 3). The values represent the mean of two replicate measurements

The sliced potatoes treated using the optimized condition described above and the untreated sliced potatoes as a control were fried at 170 °C for 90 s. The contents of both fried samples were extracted with 75 % ethanol and analyzed by GC/MS for acrylamide detection. As shown in Fig. 4, the acrylamide content of fried potatoes treated decreased to below 20 % of that of the untreated fried potatoes. These results suggested that the suppression of acrylamide might be correlated with the reduction of L-Asn caused by the BAsnase treatment combined with the optimized pretreatments. Consequently, the treatment using BAsnase with the optimized pretreatments was found to be very effective to suppress the formation of acrylamide in potato chips.

## Discussion

Since the high levels of acrylamide in heat-processed food products were discovered in 2002, mechanism of acrylamide formation during food processing and effective treatment for mitigation of acrylamide have been the focus of intense research interest. Using ^15^N-labeled L-Asn, Becalski et al. proposed that L-Asn could be the main precursor for acrylamide formation (Becalski et al. 2003). It has been clarified that L-Asn is used to form the backbone of acrylamide and that reducing sugar, such as glucose, is needed for the formation of an intermediate, which is converted into a decarboxylated Amadori product, the precursor of acrylamide (Zyzak et al. 2003; Yaylayan et al. 2003).

On the other hand, the following recipes and strategies to reduce acrylamide formation have been proposed: changing the time and temperature for heating, controlling the level of reducing sugars and L-Asn content by blanching and immersing into water, and adjusting the pH as described in the “Introduction”. Some reports have indicated that immersing and blanching potato strips as a pretreatment were effective to suppress acrylamide formation (Pedreschi et al. 2007; Ishihara et al. 2006). It has been reported that simple immersing treatment of the potato strips in water for 120 min resulted in approximately 30 % reduction of acrylamide formation and blanching them at 50 °C for 80 min resulted in the 90 % reduction of acrylamide content (Pedreschi et al. 2007). However, these treatments also decreased the amounts of the other amino acids in addition to L-Asn. According to our estimation, these treatments resulted in at least 70 % decrease of the other amino acids such as L-Glu, L-Asp, and L-Gln as well as the reduction of significant amount of L-Asn (data not shown). Some researchers have suggested that addition of microbial asparaginase from *E. coli* to potato mash and dough during mixing could be effective to mitigate acrylamide formation in final products (Zyzak et al. 2003; Amrein et al. 2004). However, the asparaginase used in these studies was from *E. coli* and the enzyme from food-grade microorganism is desirable for usage in food processing. Treatments combining asparaginase from *A. oryzae* and *B. sutilis* B11-06 with immersing and blanching have been reported to be more effective to reduce acrylamide than simple immersing/blanching treatments (Pedreschi et al. 2008; Hendriksen et al. 2009; Jia et al. 2013). These studies proved the effectiveness of combination of immersing/blanching with microbial asparaginase as a pretreatment.

**Fig. 5:**
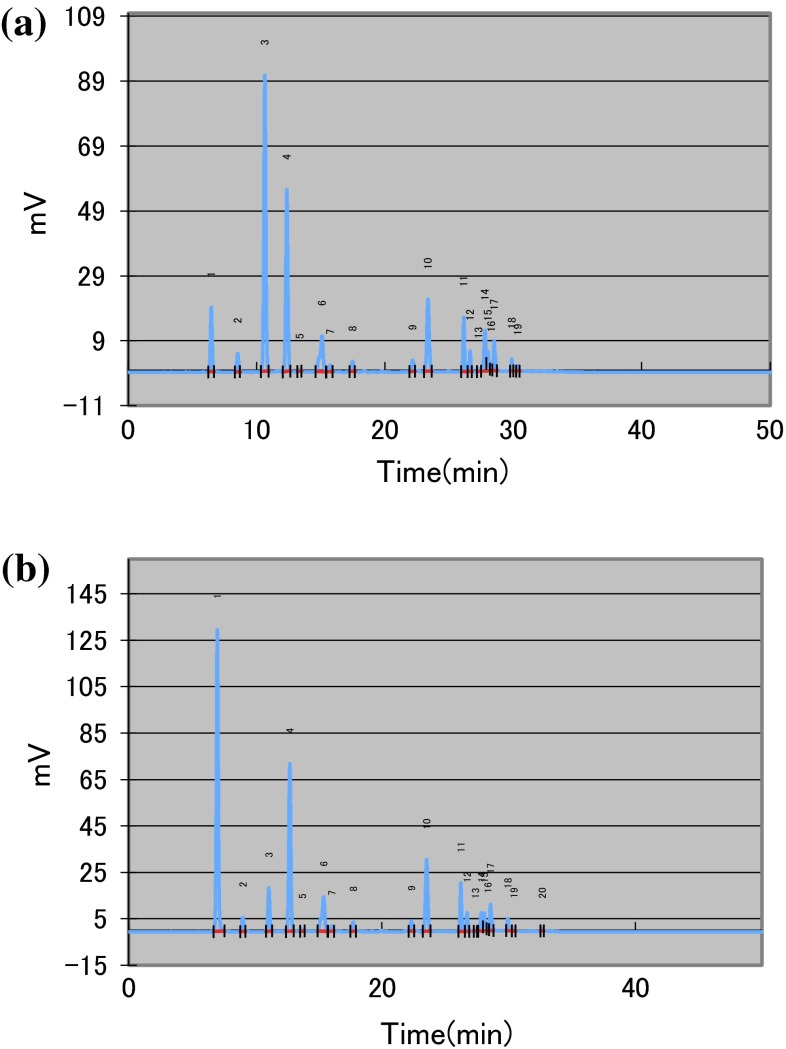
Representative HPLC chromatograms showing the major amino acid profiles of the sliced potato extracts **a** before BAsnase treatment and **b** after BAsnase treatment under optimal conditions. The numbers *1*, *2*, *3*, *4*, *6*, *10*, and *11* indicate aspartic acid, glutamic acid, asparagine, glutamine, arginine, valine, and phenylalanine, respectively. Amino acid concentration of each amino acid was calculated as follows: **a** Asp (0.89), Glu (0.35), Asn (4.93), Gln (3.08), Arg (1.03), Val (0.77), Phe (0.40) (mM); **b** Asp (5.03), Glu (0.32), Asn (1.04), Gln (3.78), Arg (1.04), Val (0.97), Phe (0.53) (mM)

BAsnase is the enzyme from *B. subtilis*, a food-grade microorganism like *A. oryzae* and *Arthrospira platensis* (Prihanto and Wakayama 2014). Because our procedure excludes immersing and blanching in water, it could significantly decrease L-Asn content with minimum loss of the other contents in the sample (Fig. 5). This method preserves the content of the sample. Consequently, the formation of desired Maillard products is expected without affecting the taste and appearance of the product. Furthermore, the treatment described here might save the amount of enzyme used by directly applying or spraying the enzyme on the sliced potatoes. Recently, there is an increase in opportunity of processing the frozen potatoes formed in a stick shape. The suppression of acrylamide formation in the fried potatoes could be expected by treating potatoes with BAsanase after thawing the frozen potatoes with a feeling similar to preprocessing a dish with a seasoning. In this case, all one has to do is to replace a seasoning with BAsnase. Enzyme treatment could become greatly efficient even just drying potatoes in an oven after thawing them. Therefore, the proposed method in this study can be useful at home or at first food shop and be low in cost because simple cooking instruments for household use are needed except for BAsnase.

It has been reported that treatment of potatoes with 3–7 % NaCl before frying resulted in the improvement of the quality of french-fries, the characteristic features being as follows: reduced oil uptake, increased hardness, and sensory texture quality (Bunger et al. 2003). BAsnase is a high salt-tolerant enzyme (Onishi et al. 2011). Using high salt-tolerant BAsnase for treatment of potatoes prior to frying will result in fried potatoes with a higher safety and quality. Therefore, our method will contribute to processing of the agricultural products with high safety and quality.
